# Exploring the recycled water acceptance based on the technological perspective of UTAUT2: a hybrid analytical approach

**DOI:** 10.3389/fpsyg.2024.1384635

**Published:** 2024-06-18

**Authors:** Xiao-Yu Xu, Yi-Bo Hu, Ya-Xuan Gao, Qing-Dan Jia

**Affiliations:** ^1^School of Economics and Finance, Xi'an Jiaotong University, Xi'an, Shaanxi, China; ^2^School of Business, Xi'an International University, Xi'an, Shaanxi, China; ^3^School of Economics and Management, Harbin Institute of Technology, Shenzhen, China

**Keywords:** recycled water acceptance, extended Unified Theory of Acceptance and Use of Technology (UTAUT2), mixed-method, structural equation modeling (SEM), artificial neural network (ANN)

## Abstract

**Introduction:**

The development of advanced sewage technologies empowers the industry to produce high-quality recycled water, which greatly influences human’s life and health. Thus, this study investigates the mechanism of individuals’ adoption of recycled water from the technology adoption perspective.

**Methods:**

Employing the mixed method of structural equation modeling and artificial neural network analysis, we examined a research model developed from the extended Unified Theory of Acceptance and Use of Technology (UTAUT2) framework. To examine the research model, this study employs a leading web-survey company (Sojump) to collect 308 valid samples from the residents in mainland China.

**Results:**

The structural equation modeling results verified the associations between the six predictors (performance expectancy, effort expectancy, social influence, facilitating conditions, environmental motivation, and price value), individuals’ cognitive and emotional attitudes, and acceptance intention. The artificial neural network analysis validates and complements the structural equation modeling results by unveiling the importance rank of the significant determinants of the acceptance decisions.

**Discussion:**

The study provides theoretical implications for recycled water research and useful insights for practitioners and policymakers to reduce the environmental hazards of water scarcity.

## 1 Introduction

Due to climate change, the size of cities, and population expansion, water shortages are becoming imperative issues that need to be addressed (Cuadrado et al., 2017; Wang and Tian, 2023). The recycled water projects are regarded as an effective and sustainable solution guaranteeing an alternative water supply (Hou et al., 2021a; Liu et al., 2022a; Maier et al., 2022). Despite recycled water is considered as one of the most imperative global resources facing environmental challenges, as for now, there are still relatively few water recycling projects underway, and recycled water is still rejected and ostracized by the worldwide people. Thus, exploring public acceptance of recycled water is essential to the successful implementation of water reuse projects and promoting the sustainable development of society and the economy (McClaran et al., 2020; Gómez-Román et al., 2021; Li et al., 2021).

Nowadays, advanced sewage technologies empower the industry to produce high-quality recycled water for irrigating, cleaning, and drinking water (Hou et al., 2021a; Li and Roy, 2021). In other words, recycled water is the product of sewage treatment technology (Hosny et al., 2016). Hence, the adoption of recycled water can be understood as the adoption of a technological product. Researchers have highlighted the importance of a technology adoption perspective since people’s resistance to recycled water is often due to their worries regarding the risks and quality of the technologies producing and supplying recycled water (Garcia-Cuerva et al., 2016). A technology adoption perspective not only enables researchers to comprehensively investigate the factors influencing people’s acceptance of a technological product but also highlights residents’ perceptions of the technical features of recycled water (Liu et al., 2018).

Importantly, people’s decision-making processes toward a technological product are complex, which is reflected in three aspects of our research context. First, the decision-making process might be influenced by various factors, such as features of sewage treatment technology, the infrastructure to supply recycled water, government policy and guidance, the social environment (social norms), and the price of recycled water (Chen et al., 2013; Glick et al., 2019; Hartley et al., 2019; Leong and Lebel, 2020; Iftekhar et al., 2021; Hopson and Fowler, 2022; Vila-Tojo et al., 2022; Zhang et al., 2022). Hence, the investigation of the acceptance of recycled water should embrace the significant factors from a comprehensive perspective. Second, people often develop behavioral intentions based on their cognitive understanding and emotional attitudes (Wester et al., 2016; Li et al., 2022). For example, people may have negative affection toward recycled water (e.g., disgust) due to their stereotype of recycled water, or positive affection, due to their environmental awareness (Hou et al., 2021a; Vila-Tojo et al., 2022). Therefore, both the cognitive and emotional mechanisms should be carefully considered when investigating the adoption of recycled water. Third, the analytic methods adopted should be carefully designed to unveil the complexity of decision-making, since the relationships between the influence factors and behavior might not necessarily be linear (Chong, 2013). To conclude, the investigation of public acceptance of recycled water should employ a comprehensive model from a technology adoption perspective for integrating multiple factors and both cognitive and emotional mechanisms with a hybrid analytical design.

Although prior studies have deepened our understanding of the determinants and mechanisms of public acceptance toward recycled water (Fielding et al., 2018). Three research gaps remain in the literature. First, most extant literature has identified psychological, social, and sociodemographic factors to interpret people’s acceptance from the perspective of social psychology (Nancarrow et al., 2009; Dolnicar et al., 2011; Chen et al., 2013; Schultz and Fielding, 2014; Savchenko et al., 2019; Hou et al., 2020, 2021b; Leong and Lebel, 2020; Ding et al., 2022). There still lacks of knowledge taking the perspective of technology adoption to investigate the individuals’ adoption intention. Second, previous study has mainly concentrated on a single aspect to explore the influence on recycled water acceptance, such as individuals’ perceptions (Li et al., 2022), social influence (Leong and Lebel, 2020), or emotional factors (Ding and Liu, 2021). Little research has explored the relationships between the determining factors and the cognitive and emotional reactions to illustrate the complexity of people’s decision-making processes toward recycled water (Wester et al., 2015; Mahmoud-Elhaj et al., 2020). Thus, there still lacks the knowledge regarding how the dual mechanisms—cognitive and emotional—determine recycled water adoption. Third, prior studies mainly adopted a single analysis technique with regression analysis or structural equation modeling (SEM). However, these methods assume the linear and compensatory relationship between constructs, which may oversimplify the complex individuals’ decision-making processes (Wester et al., 2016). Thus, in a new technologically empowered research context, individuals’ acceptance behavior may be more complex. Multiple analytical approaches should be employed to examine the relationships with a higher predictive power and rank the importance of antecedents to identify the most significant drivers in influencing individuals’ acceptance decisions.

Addressing the identified research gaps, first, this study aims to apply the extended Unified Theory of Acceptance and Use of Technology (UTAUT2) theoretical framework to interpret individuals’ decision-making processes regarding recycled water from the technology adoption perspective (María et al., 2022). The employment of UTAUT2 offers theoretical innovation in the literature, since this framework distills the critical factors toward a technological product from consumers’ perspectives (Venkatesh et al., 2012), which is worth contextualizing UTAUT2 to unveil people’s recycled water acceptance. Second, as one of the pioneer works, this study endeavors to provide new knowledge by addressing both cognitive and emotional attitudes to fully explore individuals’ perception responses in determining the acceptance of recycled water. Third, this study takes innovative research approaches by employing both SEM and artificial neural network (ANN) analysis. The hybrid analytical approach not only can verify the hypothesized relationships in influencing recycled water acceptance, but also can reveal the importance rank of critical factors with a supplementary and reinforced perspective (Le and Li, 2022). This study aims to offer significant methodological implications which can provide a comprehensive and robust exploration into the underlying mechanism of residents’ acceptance processes toward recycled water.

## 2 Theoretical background and research hypotheses

### 2.1 Recycled water

With the increasing populations and climate change, water scarcity has become an imperative global issue influencing people’s lives and health (Liu et al., 2022a). As one of an ideal alternative solution, recycled water helps people conserve valuable freshwater resources. Recycled water is the wastewater that is processed and purified to remove contaminants and impurities and thus enabling it suitable for use. Recycled water is essential in sustainable water management and conservation, providing an alternative source of water for various needs, such as industrial uses, agricultural and landscape irrigation, urban and recreational uses, and potable reuse, etc. In this study, we focus on the application of recycled water in personal life, particularly in households and daily activities, such as plant irrigating, car washing, toilet flushing, fire suppression, laundry, and street cleaning, etc. Since the incorporation of recycled water in personal life is vital in water conservation, ensuring the sustainability of water supply, and ensuring long-term water security to support the sustainable development of human society. Thus, the investigation of the public adoption intention toward recycled water has been a hot topic worldwide.

Despite recycled water is regarded as the most significant global resource facing environmental issues, as for now, there are still relatively few water recycling projects underway, and recycled water is still repugnant and ostracized by the public worldwide (Liu et al., 2022a; Vila-Tojo et al., 2022). For example, Australia, the US, Saudi Arabia, and China all face the problem of low public acceptance of recycled water (Gao et al., 2019; Barnes et al., 2021; Hou et al., 2021b; Santos et al., 2022). For example, the implementation of direct potable reuse projects is repugnant and ostracized by the public, with few initiatives currently in operation globally (Santos et al., 2022). In Australia, the initiatives to implement recycled water systems did not achieve intended outcomes, with over 60% of the populace opposing the initiative. A parallel situation unfolded in San Diego County, where substantial financial commitments to develop recycled water infrastructure met with extensive public opposition. Meanwhile, in China, prevailing societal biases continue to challenge the acceptance of recycled water. In addition, existing news and investigation reports also widely point out that the low public adoption intention has become an imperative issue hindering the development and efficient utilization of recycled water at present. Considering the vital role of recycled water in alleviating water resource shortages and protecting the water environment and water ecology, the public adoption intention toward recycled water is the key to address the water shortage issue.

Various studies in recent years all proposed that public perceptions and acceptance toward recycled water is still a timely research topic and are worthy to explore. For example, Gao et al. (2019) highlighted the resident’s perception of recycled water as the foundation to promote their recognition, acceptance, willingness, and hope to use recycled water. Li et al. (2022) focused on the residents’ acceptance of recycled water and explored the critical social factors in influence their attitudes and usage intentions toward recycled water. Vila-Tojo et al. (2022) also suggested that it is critical to understand the public perceptions regarding why acceptance or rejection of recycled water to implement the use of recycled water. Liu et al. (2022b) conducted a literature review and reviewed the studies exploring public acceptance of recycled water. They indicated that recycled water remains controversial and is rejected as water from wastewater treatment by the public. Thus, the exploration of the critical factors influencing public water acceptance is imperative and valuable.

### 2.2 The acceptance of recycled water

Existing literature mainly employed three perspectives to investigate the public acceptance of recycled water (Fielding et al., 2018; Smith et al., 2018; Hou et al., 2021a; Reddy et al., 2023). First, many researchers adopted the models and theories from the field of individual’s psychology. Gullberg et al. (2023) proposed that acceptance of recycled water is associated with individual’s different psychosocial aspects such as underlying attitudes, personal norms, and values. Chen et al. (2013) especially highlighted the relationships between various psychological variables and the acceptance of recycled water. Wester et al. (2016) focused on the perspective of individual’s psychology of recycled water and empirically verified the influence of psychological factors on people’s recycled water usage willingness. Similarly, Vila-Tojo et al. (2022) suggested that a psychological framework, namely the perceptive-axiological model, is useful for examining the effect of scarcity diagnosis on people’s acceptance of recycled water.

Second, several studies highlighted the effect of social influences on the acceptance of recycled water, such as the influence of government, environmental institutions, group members, social networks, etc. For example, Liu et al. (2022a) explored the influence of social norms in influencing individual’s willingness to use recycled water. Fu et al. (2022) proposed that the degree of human contact significantly impacted the individual’s negative stereotypes toward recycled water. In-group identity and social norms (conformity) may enhance the impact of information on individuals’ perceptions of recycled water (Schultz and Fielding, 2014; Leong and Lebel, 2020). Similarly, researchers have found that the influence of governments and water utilities can facilitate residents’ positive perceptions of the information about reused water (Ross et al., 2014; Hou et al., 2020, 2021b; Ding et al., 2022). Thus, various studies in recent 3 years all proposed that public perceptions and acceptance toward recycled water is still a timely research topic and are worthy to explore (Barnes et al., 2023; Tanner et al., 2024).

Third, prior literature has investigated the relationships between sociodemographic features and individuals’ acceptance of recycled water. For example, Abubakar and Mu'azu (2022) examined the effect of educational factors on public acceptance of recycled water use. Tshililo et al. (2024) also confirmed that higher levels of education were more likely to accept reclaimed water. Similarly, Li et al. (2020) employed a meta-analysis to investigate the role of various social demographic factors in influencing individuals’ re-cycled water acceptance.

Though existing studies provide rich and valuable understandings of people’s acceptance of recycled water, little research adopted the perspective of technology adoption to interpret the acceptance of reused water. Only Liu et al. (2018) applied the technology acceptance model (TAM) to investigate people’s attitudes and acceptance intention toward recycled water technology. A technology adoption perspective does not suggest the investigation of merely technical features of an object, but suggests understanding the adoption of an object (e.g., recycled water) as the adoption of a technological product, and thus examining how people’s attitudes toward recycled water as a technological product are influenced by a variety of factors. In other words, accepting recycled water depends on individuals’ perceptions and understandings of such a new technological product rather than the technology itself.

Additionally, the present research findings seem insufficient to reveal the complexity of the people’s decision-making process of accepting recycled water, since most of the extant studies merely investigated the mechanisms from a single aspect, such as examining social or psychological factors. For example, Dolnicar et al. (2011) focused mainly on the associations between psychological constructs and public acceptance of recycled water. Leong and Lebel (2020) explored the role of conformity as a social norm in increasing residents’ approval of recycled water reuse. Hartley et al. (2019) employed a formal mathematical model to explore how national policies, water facilitations, and the interaction among the residents to promote or impede the acceptance of recycled water. In addition, existing literature has mainly adopted a single research design, such as structural equation modeling analysis, regression analysis, and experiment study (Gao et al., 2019; Hou et al., 2020, 2021a; Ding and Liu, 2021; Ding et al., 2022). For example, Hou et al. (2021b) applied a structural equation model to examine the effects of information disclosure on public acceptance of recycled water. Garcia-Cuerva et al. (2016) analyzed experimental data using a linear regression model to explore the impacts of several predictors on residents’ willingness to accept recycled water. However, the complexity of individuals’ decision-making processes may be oversimplified by merely adopting a linear analysis (Chong, 2013). It requires employing a hybrid analytical approach with a complementary analytical approach to address the limitation of linear analysis. To conclude, the investigation of the comprehensive antecedents under a solid theoretical framework and the application of the hybrid analytical design may unveil the complexity of people’s recycled water adoption with a higher predictive power.

### 2.3 The extended Unified Theory of Acceptance and Use of Technology (UTAUT2)

Through a review and a comprehensive synthesis of eight prominent theories employed in technology adoption research, Venkatesh et al. (2003) proposed the Unified Theory of Acceptance and Use of Technology (UTAUT) to interpret individual behavioral intention to accept and use new technologies, primarily in organizational contexts. Four constructs grouping similar earlier constructs were embraced in this model, including performance expectancy, effort expectancy, social influence, and facilitating conditions. Since its initial publication, UTAUT has been applied as a baseline model to explain the acceptance and use of various technologies in different contexts. Due to the rise of consumer technologies, the UTAUT model must be extended to the consumer context emphasizing the salient factors (e.g., hedonic value) significantly impacting their technology acceptance. Therefore, three additional factors, including hedonic motivation, price value, and habit, were integrated into the initial UTAUT and formed the new extended model, namely the extended Unified Theory of Acceptance and Use of Technology (UTAUT2) (Venkatesh et al., 2012). Researchers have applied UTAUT2 to explore consumers’ acceptance of focal technology and highlighted that UTAUT2 has a substantially more predictive capacity than UTAUT (Venkatesh et al., 2016). Therefore, UTAUT2 has been widely employed to understand consumers’ behaviors toward various new technology.

The UTAUT2 has been successfully applied and verified in the exploration of a variety of contexts, such as mobile app usage (Hew et al., 2015; Chang et al., 2019; Alalwan, 2020), internet banking adoption (Baptista and Oliveira, 2015; Alalwan et al., 2018), and smartphone adoption (Venkatesh et al., 2012; Shaw and Sergueeva, 2019). Previous researchers applied various extended versions of UTAUT2 to explore consumers’ acceptance and use of technology, such as integrating new exogenous, endogenous, moderating, outcome, and mediating variables into the initial model, and extending the theory with new external and internal mechanisms (Venkatesh et al., 2016; Alalwan, 2020). Accordingly, UTAUT2 has been widely acknowledged as a comprehensive and robust theory to explore consumers’ decision-making process of new technology with identified relevant predictors in specific contexts. In the context of recycled water, each person can be understood as a water consumer, and recycled water is the product of sewage technology. Thus, people’s acceptance of recycled water can be interpreted as the water consumers’ adoption of the technological product, namely recycled water. As indicated by Liu et al. (2018), it is necessary to understand the process of residents’ recycled water technology acceptance so that water utilities can find the most effective strategies to promote water reuse. Hence, considering the critical role of UTAUT2 in comprehending individuals’ underlying decision mechanisms, we applied UTAUT2 as the theoretical framework to explore the recycled water consumers’ decision-making processes.

UTAUT2 is valuable to identify the critical environmental factors related to the prediction of behavioral intention to use a technology from the technological perspective. While it limits in explaining how these environmental factors influence individual information processing. Mindsponge theory posits that individuals evaluate their acceptance of information from the external environment based on value judgments and assessment (Vuong, 2022, 2023). Prior studies have widely applied the mindsponge theory to explore how individuals continuously absorbing and excluding information to provide a more logical interpretation about the effects of environmental factors on individual’s behavior (Lu et al., 2023; Mantello et al., 2023). The Mindsponge theory provides a conceptual framework that explains individual’s information processing mechanisms and details how people are influenced by the environmental factors. Thus, the integration of UTAUT2 and mindsponge theory provides a comprehensive explanation of what and how various factors influence individual’s cognitive and emotional attitudes and their reused water adoption intentions.

Seven constructs are included in UTAUT2, namely performance expectancy, effort expectancy, social influence, facilitating conditions, price value, hedonic motivation, and habit (Venkatesh et al., 2012). The integration of these constructs has comprehensively captured the critical factors determining recycled water acceptance (Dolnicar et al., 2011; Fielding et al., 2018; Liu et al., 2018; Hartley et al., 2019). While recycled water is not a product designed for hedonic purposes such as entertainment or fun, its hedonic value is marginal. Instead, people’s environmental motivations, such as environmental awareness, have been suggested as a significant predictor in influencing recycled water acceptance (Moya-Fernández et al., 2021). Hence, environmental motivation rather than hedonic motivation is more suitable to be investigated in this study. Moreover, since the marketing promotion of recycled water projects is still embryonic, existing studies mainly focus on investigating people’s initial understanding and acceptance intention. In other words, individuals are very unlikely to develop a habit of using recycled water daily. Hence, the construct habit proposed in the original UTAUT2 is not included in our research model.

In addition, both the cognitive and emotional mechanisms may exert important influences on recycled water adoption (Gao et al., 2019). This study integrated cognitive and emotional attitudes into the research model to explore the dual mechanism in determining recycled water acceptance.

The following sections will elaborate in detail on how the hypotheses of constructs were developed and justified.

## 3 Research model and hypotheses development

**Figure 1:**
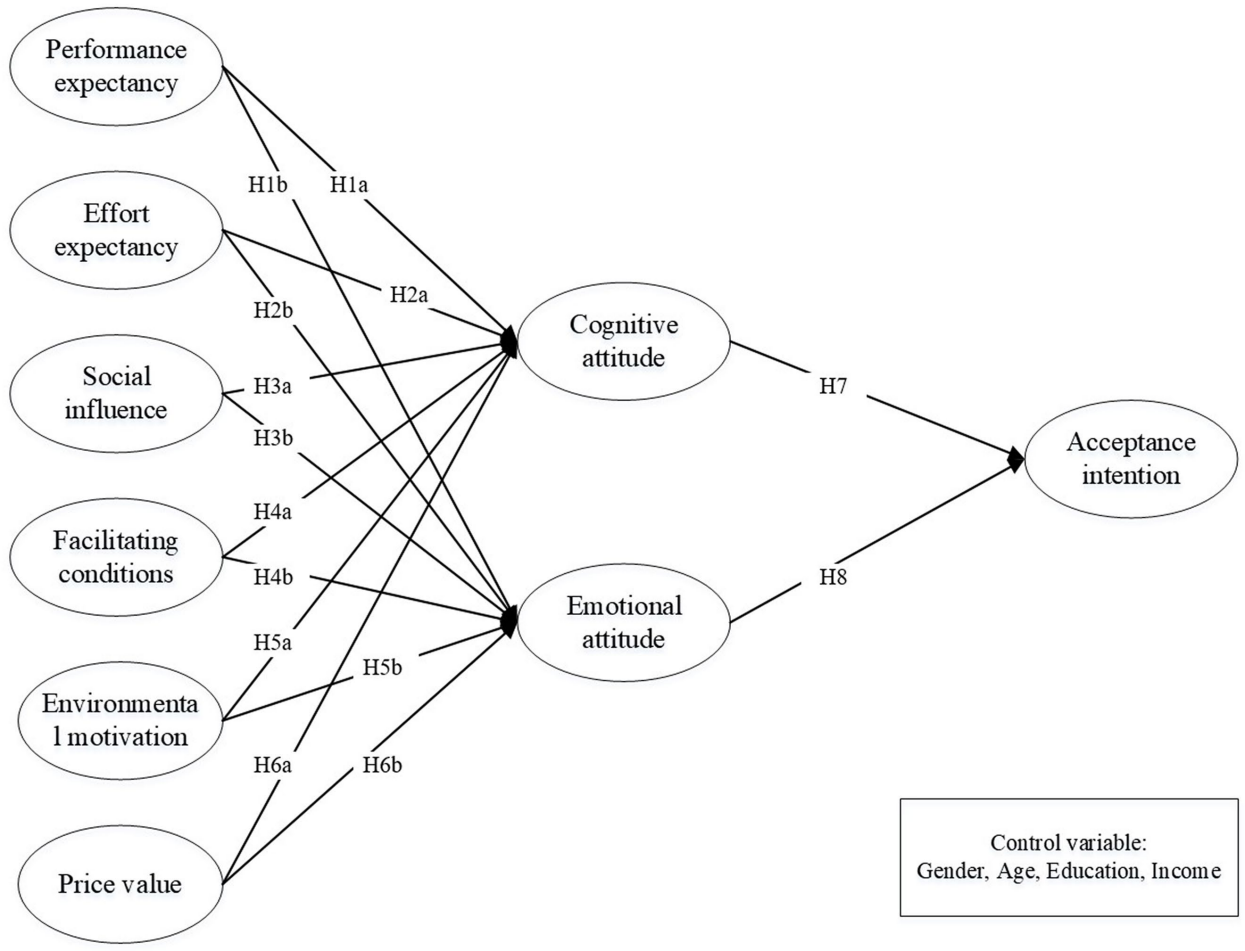
Research model.

To explore individuals’ decision-making processes of recycled water, a research model was established to examine the impact of five factors that are determined in original UTAUT2 and one specific factor identified from the context of public acceptance of recycled water. The research model is presented in Figure 1. Cognitive and emotional attitudes are two crucial distinct concepts in psychology in forming an individual’s overall attitude toward something, influencing individual’s behavior and decision-making toward recycled-water (Gao et al., 2019). Cognitive attitude refers to the individual’s degree of the understanding about recycled water, which is regarded as the personal evaluation toward an object (Gao et al., 2019; Li et al., 2022). Emotional attitude captures consumers’ feelings, which refers to the psychological state of an individual’s affective attitudes about recycled water (Sabates, 2022). This study embraces the two dimensions of attitude to examine how the antecedents included in extended UTAUT2 impact individuals’ attitudes and acceptance intentions.

### 3.1 Performance expectancy

Performance expectancy refers to the extent of consumers believe that the adoption of technology will be advantageous in carrying out specific activities (Venkatesh et al., 2012). Recycled water is regarded as a reliable source used for satisfying the needs of urban life, industrial, and agricultural purposes, and has the potential benefits for future generations due to the reduction of over-extraction from natural sources (Hartley et al., 2019). When individuals perceive the usefulness and benefits associated with using recycled water, they are likely to realize the importance and value of recycled water. Moreover, several researchers have identified that people would develop positive emotional reactions (e.g., satisfaction) toward recycled water because of the benefits of its usage (Hurlimann et al., 2008). Hence, we propose that:

*Hypothesis 1a*: Performance expectancy is positively associated with cognitive attitude.

*Hypothesis 1b*: Performance expectancy is positively associated with emotional attitude.

### 3.2 Effort expectancy

Effort expectancy refers to the extent of ease perceived by individuals to use recycled water (Venkatesh et al., 2012). As indicated by Liu et al. (2008), if residents perceive the use of recycled water as easy and convenient, their attitudes toward it would be more positive. On the one hand, due to the ease and convenience of using recycled water, people can access recycled water effortlessly and may have sufficient opportunities to understand and evaluate recycled water comprehensively. In addition, when individuals perceive the ease of using recycled water, they are likely to believe they can use recycled water without any barriers, making them feel relaxed, relieved, and confident about water reuse (Wester et al., 2016). Hence, we proposed that:

*Hypothesis 2a*: Effort expectancy is positively associated with cognitive attitude.

*Hypothesis 2b*: Effort expectancy is positively associated with emotional attitude.

### 3.3 Social influence

Social influence refers to the degree to which individuals perceive that important others (such as family and friends) believe they should use recycled water (Venkatesh et al., 2012). Previous researchers have found that people’s attitudes and acceptance of recycled water are influenced by the opinions and behaviors of others (Dolnicar et al., 2011; Chen et al., 2013; Leong and Lebel, 2020). If recycled water is widely supported and adopted by friends and families, residents have the opportunities and channels to understand recycled water comprehensively, and thus they will be convinced of the value and importance of recycled water; and are inclined to be affected by others’ perceptions toward an object of using new technological products (Alalwan, 2020). Hence, we proposed that:

*Hypothesis 3a*: Social influence is positively associated with cognitive attitude.

*Hypothesis 3b*: Social influence is positively associated with emotional attitude.

### 3.4 Facilitating conditions

Facilitating conditions refer to the individuals’ perceptions toward the resources and support available to use recycled water (Venkatesh et al., 2012). Several researchers have highlighted the positive association between facilitating conditions and people’s perceptions of water reuse (Hartley et al., 2019). When people are exposed to reliable and transparent information, they can understand and evaluate recycled water comprehensively (Ross et al., 2014). Meanwhile, if individuals feel control over the source and quality of recycled water, they would consider it a safe and reliable water source, and thus their emotional responses would be positive (Nancarrow et al., 2009; Craddock et al., 2021). Hence, we proposed that:

*Hypothesis 4a*: Facilitating conditions are positively associated with cognitive attitude.

*Hypothesis 4b*: Facilitating conditions are positively associated with emotional attitude.

### 3.5 Environmental motivation

Environmental motivation is defined as a consumer’s awareness of environmental protection and an ecological desire to use recycled water (Venkatesh et al., 2012). The positive relationships between residents’ environmental awareness and their attitudes and behaviors toward recycled water have been confirmed (Hou et al., 2020; Moya-Fernández et al., 2021). If people are aware of water and environmental conservation, they are likely to regard recycled water projects as essential for addressing water scarcity and protecting the environment and develop a positive perception of water reuse. Additionally, using recycled water is viewed as a pro-environmental action, and individuals motivated by environmental awareness are likely to feel proud of their use of recycled water (Hou et al., 2021b). Hence, we proposed that:

*Hypothesis 5a*: Environmental motivation is positively associated with cognitive attitude.

*Hypothesis 5b*: Environmental motivation is positively associated with emotional attitude.

### 3.6 Price value

The price value refers to the cognitive tradeoff between the perceived benefits and costs of adopting recycled water (Baptista and Oliveira, 2015). Price advantages are vital to public acceptance of recycled water. The reasonable price of recycled water will promote the positive perceptions of the people who prefer economic benefits (Zhang et al., 2022). Accordingly, they are likely to evaluate water reuse as a cost-effective way to satisfy their demands, and hence enhance their cognitive attitudes toward recycled water (Chen et al., 2013; Garcia-Cuerva et al., 2016). Additionally, the economic benefits have great influences on public emotional feelings (e.g., satisfaction) about recycled water. Individuals’ emotional reactions will be positive due to the price value (Hurlimann et al., 2008). Hence, we proposed that:

*Hypothesis 6a*: Price value is positively associated with cognitive attitude.

*Hypothesis 6b*: Price value is positively associated with emotional attitude.

### 3.7 Acceptance intention

Acceptance intention is conceptualized as the extent to which the customer’s willing to accept recycled water (Venkatesh et al., 2012). The positive perceptions and attitudes toward recycled water have been confirmed as the key drivers for acceptance (Dolnicar et al., 2011; Fielding et al., 2018). When people’s cognitive attitudes toward recycled water are positive, they will make comprehensive and deliberative evaluations of water reuse and understand its value and importance, hence they will be willing to accept it (Gao et al., 2019). In addition, the positive emotional attitudes imply that the feelings about using recycled water are honorable and relieved, and thus facilitate people to make positive decision-making (Chen et al., 2013). Hence, the following hypotheses are proposed:

*Hypothesis 7*: Cognitive attitude is positively associated with acceptance intention.

*Hypothesis 8*: Emotional attitude is positively associated with acceptance intention.

## 4 Materials and methods

### 4.1 Measurement development

The measurements of the constructs used in this study were primarily drawn from previous research, with some adjustments made to suit our research context. The items for measuring performance expectancy (PE) and effort expectancy (EE) were adopted from the study of Venkatesh et al. (2012), Khan et al. (2017), and Liu et al. (2018). This study utilizes the items from Venkatesh et al. (2012) to measure social influence (SI). The items for measuring facilitating conditions (FC) were adapted from the research of Brown and Venkatesh (2005) and Venkatesh et al. (2012). Environmental Motivation (EM), according to the features of recycled water use context, stresses individuals’ environmental awareness. The measures for environmental motivation were taken from Venkatesh et al. (2012) and Razak et al. (2014). We developed the items from Venkatesh et al. (2012) to measure price value (PV). The measures of cognitive attitude (CA) and emotional attitude (EA) were modified by Chiu et al. (2019) and Zhang et al. (2021). Finally, the items for acceptance intention (AI) were modified by Venkatesh et al. (2012) and Khan et al. (2017). The final measurement items are shown in Supplementary Appendix A. We employed 5-point Likert scale, ranging from 1 (indicating strong disagreement) to 5 (indicating strong agreement) for assessing all the items.

To ensure the validity of the measurements, a panel discussion was conducted. We invited ten experts including five researchers in the research field of recycled water and five practitioners in the recycled water industry. Based on their feedback, we revised the wording, language, and expression issues in the questionnaire to ensure the readability and understandability of our questionnaire. In addition, a small-scale pilot test was conducted by inviting 150 respondents with recycled water usage experience to validate the reliability and validity of our questionnaire.

### 4.2 Sampling and data collection

We employed one of the most famous survey company that is widely used by the public in China, Sojump, to collect our empirical data. As the largest developing country and one of the countries with the largest populous, China is a representative country facing the water shortage problem. Thus, it is appropriate to apply China as a typical study site to explore the public acceptance intention toward recycled water in addressing the water shortage issue. Moreover, China is a typical country where the public remains in the “wait-and-see” phase toward the recycled water usage, and the public lack of knowledge and awareness regarding recycled water. Considering the research focus of this study is exploring users’ acceptance intentions of recycled water, China is an appropriate research context to provide evidence and references for studying this focal phenomenon in other countries.

Following the three-step sampling process indicated by Bhattacherjee (2012), we determined the target population, chose a sampling frame, and distributed the questionnaires based on random sampling. This study employed a leading web-survey website^1^ to collect the empirical data from the urban residences in China during September 3, 2023 to September 20, 2023. Considering it is critical to ensure the generalizability of results, we adopted random sampling in that all possible subsets of a population were given an equal probability of being selected. To ensure the respondents fully understand our research context, we first presented the definition and description of recycled water at the beginning of the online questionnaire. The attention check questions encompass both contradictory queries and duplicated questions. Are incorporated in the questionnaire to ensure the validity of the responses. Monetary rewards were provided to motivate people participate in the survey. Each participant who filled out the questionnaire could receive a compensation of 5–10 RMB (about 1 ~ 1.5 USD). Additionally, to further encourage participation, a lottery incentive was introduced, which approximately 20% of the respondents were randomly selected to receive. Finally, a total of 356 participants engaged in the survey. After the data screening, a total of 308 responses were confirmed as valid for the subsequent data analysis.

The demographic information of respondents (see Supplementary Appendix B) shows that 52.3% of the respondents are females, and 44.7% are males, which is similar to existing studies related to recycled water (McClaran et al., 2020). In addition, most of the respondents are aged from 20 to 35 (74.4%), 62.7% of the respondents are undergraduates, and the per month income of most respondents is below 8,000. The age, educational degree, and income distribution of our sample comply with that in the existing literature (Buyukkamaci and Alkan, 2013; Fielding et al., 2018).

## 5 Data analysis and research results

Employing Smart PLS software, this study conducted the SEM analysis to examine the empirical data. A two-phase confirmatory factor analysis approach is conducted to first analyze the measurement model and then the structural model.

### 5.1 Measurement model analysis

To assess the validity measurement model, this study first examined the convergent validity used various metrics, including Cronbach’s alpha, factor loading, composite reliability (CR), and Average Variance Explained (AVE) (Chin, 1998). According to the results presented in Supplementary Appendix C, the values for both CR and Cronbach’s alpha are above the threshold of 0.7, the factor loading of all items exceed 0.7, and the AVE values are greater than 0.5. All these results verify that the satisfaction of convergent validity. Furthermore, the discriminant validity was examined by the square root of each AVE should exceed the inter-construct correlations. Based on the results presented in Supplementary Appendix D, all constructs in our measurement model demonstrate discriminant validity.

In addition, we assessed the model fit of the measurement model. The standardized root mean square residual (SRMR) should not surpass 0.08. The SRMR of study is 0.05. Therefore, these results confirm a satisfactory model fit.

### 5.2 Common method bias examination

Considering the self-report survey data may exist the common method biases (CMB) since the empirical data is from the same sources (Jakobsen and Jensen, 2015). Thus, the examinations based on procedural assurance and statistical examination were applied to ensure this study avoids the severe influence of CMB (Li et al., 2022; Lo et al., 2022).

As referred to the procedural measures (Podsakoff and Organ, 1986), the anonymity and confidentiality of the survey data ease the respondents’ concerns when answering questions, which can ensure the correctness of the answers. In addition, to ensure clarity and reduce respondents’ lack of understanding of the questions, we endeavor to apply simple sentences and precise language in our questionnaire (Liang et al., 2007).

We employed two statistical approaches to assess the potential threat of CMB. First, we conducted Harman’s single-factor test. The results revealed that the maximum covariance explained by a single factor is 29.674%, which is below the acceptable threshold of 50% (Podsakoff and Organ, 1986). Secondly, we examined the interrelationships among the constructs. The findings showed that all correlations between variables remain below the 0.9 threshold (see Supplementary Appendix E) (Bagozzi et al., 1991). Thus, based on the examination results, the CMB is not a substantial concern affecting the reliable and validity of our research results.

### 5.3 Structural model analysis

**Figure 2:**
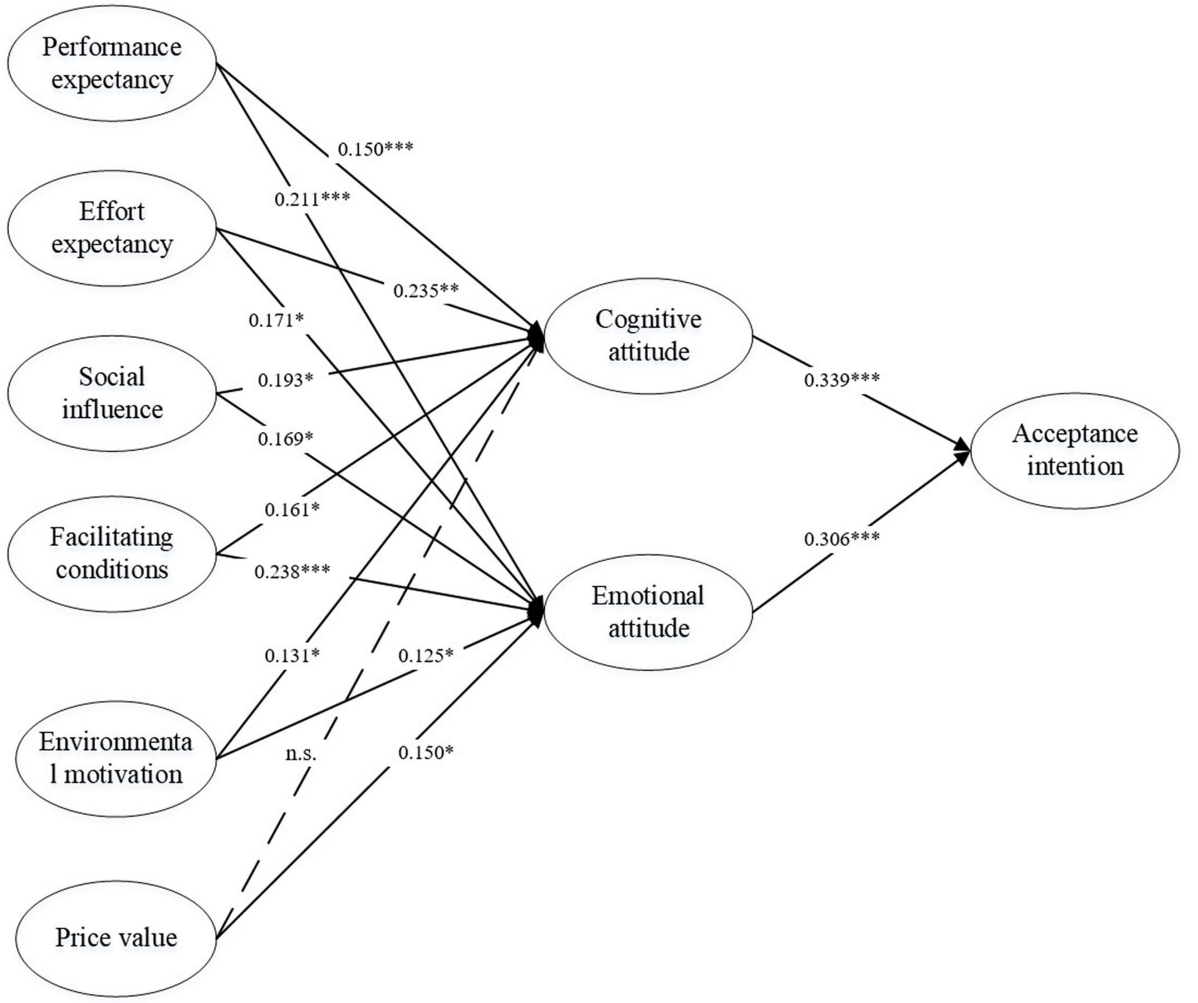
Research results.

The explanatory power and path importance of structural models were examined (see Figure 2). The explained variances for the cognitive attitude, emotional attitude, and behavioral intention are 60, 65, and 36%, respectively. In addition, SRMR was 0.05, which implies that the overall model fit is good.

Regarding the impact of the six predictors on cognitive attitude and emotional attitude, the test results support hypotheses H1a (*β* = 0.150, *p* < 0.01), H1b (*β* = 0.211, *p* < 0.001), H2a (*β* = 0.235, *p* < 0.001), H2b (*β* = 0.171, *p* < 0.05), H3a (*β* = 0.193, *p* < 0.05), H3b (*β* = 0.169, *p* < 0.05), H4a (*β* = 0.161, *p* < 0.05), H4b (*β* = 0.238, *p* < 0.001), H5a (*β* = 0.131, *p* < 0.05), and H5b (*β* = 0.125, *p* < 0.05). The beta coefficient is the degree of change in the outcome variable for every 1 unit of change in the predictor variable, which is employed to evaluate how a regression equation fits data. The results reveal that both cognitive attitude and emotional attitude are positively associated with performance expectancy, effort expectancy, facilitating conditions, environmental motivation, and habit in terms of recycled water use. In addition, it is worth noting that the relationship between price value and cognitive attitude (H6a) is not supported, while the relationship between price value and emotional attitude (H6b) (*β* = 0.150, *p* < 0.05) is supported. At last, we further examined the association between two types of people’s attitudes and behavioral intentions. The results unveil that both cognitive attitude and emotional attitude strongly affect behavioral intentions; thus, hypotheses H7 (*β* = 0.339, *p* < 0.001) and H8 (*β* = 0.306, *p* < 0.001) are supported.

### 5.4 Artificial neural network (ANN) analysis

#### 5.4.1 Artificial neural network (ANN)

In recent years, the accuracy of SEM analysis has been criticized since it assumes linear and compensatory relationships between the variables, which oversimplifies the complex and multifactor-influenced decision-making processes (Lo et al., 2022). Our research context is an emerging field that lacks exploration and incorporated complicated individuals’ perceptions toward the new technology (Chong, 2013). A robust and rigorous analytical approach should be applied to complement and validate the SEM results, which can further address the complex decision-making process toward recycled water adoption.

In recent years, artificial neural network (ANN) modeling has been widely used as a machine-learning tool to mimic the human brain and reveal the latent relationships among the data. ANN is an advanced analytical approach that involves computations and mathematics to analyze the data with a higher prediction accuracy. The input layer in an artificial neural network receives data from the outside world which the neural network needs to analyze or learn about. A hidden layer is a layer between input layers and output layers, which can capture the complex nonlinear behaviors of data more efficiently by learning and extracting the information from the training data, and further generating the weight values of the connections between the layers. Finally, the output layer transfers the information that the network has processed to the outside. In other words, the output layer gives the answer or prediction of the ANN model based on the input from the input layer.

ANN has the advantage of verifying both the linear and non-linear relationships against noise, outliers, and small sample sizes. In other words, the strength of ANN empowers it to rank the significance of input factors with a higher predictive power. Specifically, the use of activation functions such as sigmoid in ANN analysis allows ANN to capture complex relationships in the input data. Each neuron in an ANN applies a non-linear activation function to its input, transforming it before passing it on. During analysis, the network refines its understanding of these non-linear relationships by adjusting the weights and biases. This process, facilitated by backpropagation and optimization algorithms, allows the network to learn which features are important and how they interact non-linearly to affect the output. Through these mechanisms, ANNs effectively rank the importance of the antecedents by inculpating linear and non-linear relationships with higher predictive power (Leong et al., 2020).

However, the ANN analysis is a “black box” operation, which is not appropriate to examine the proposed hypothesis. Thus, the advantages and shortcomings of SEM can be naturally combined with ANN. The integration of these two analytical approaches can achieve both the hypothesis testing and prediction objectives in data analysis, and further improve the accuracy in exploring the individuals’ complex decision-making procedures.

To fully address the individuals’ decision-making process toward recycled water under the influence of diversified factors. A dual-stage SEM-ANN approach is applied in this study to first validate the hypothesized factors affecting recycled water acceptance. Second, it can further rank the importance of the verified factors and identify the most critical drivers in influencing individuals’ decision-making toward recycled water with higher prediction accuracy.

**Figure 3:**
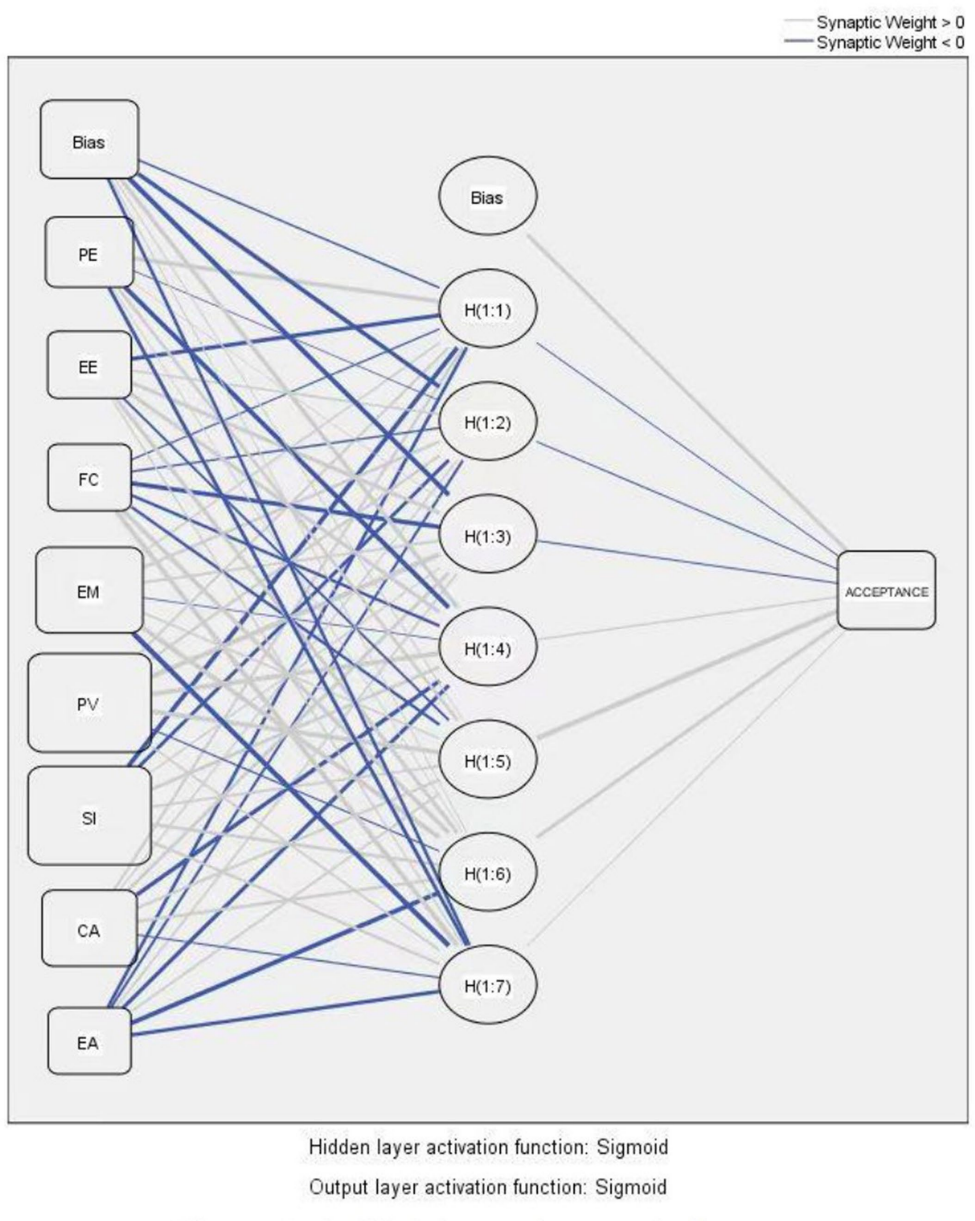
Artificial neural network diagram. PE, Performance Expectancy; EE, Effort Expectancy; FC, Facilitating Conditions; EM, Environmental Motivation; PV, Price Value; SI, Social Influence; CA, Cognitive Attitude; EA, Emotional Attitude.

Following the above notion, we first examine the proposed hypotheses by applying SEM analysis and obtained the significant determinants. Incorporating these verified significant factors, we employed them as the input neurons in the ANN model to investigate their importance rank in affecting acceptance intention to understand which factors are valuable to residents. The ANN model is shown in Figure 3.

#### 5.4.2 Validation of neural network

Employing IBM SPSS Neural Networks, this research used a commonly used feed-forward backpropagation multilayer training algorithm with a sigmoid function as the activation function for both hidden and output layers to train the ANN model, and the number of hidden neurons was generated automatically by the SPSS Neural Network algorithm (Leong et al., 2020).

To avoid the threat of overfitting, this study applied 90% data for training and 10% data for testing. And conducted the ten-fold cross-validation analysis to validate the ANN models (Sharma et al., 2021).

The predictive accuracy of the ANN model can be measured by the Root Mean Square of Error value (RMSE) which can show the average difference between the predicted values and the actual values. A lower and similar RMSE value implies a higher level of predictive accuracy and represents a great fit and forecast of the data. As shown in Table 1, the results show that the lower average RMSE values ranged from 0.089 to 0.095 for both training and testing processes (Lo et al., 2022). The average difference between training and testing RMSE values in the model is small and similar, which suggests that the ANN analysis results are reliable.

**Table 1:** Neural network validation results.

Neural network	Training	Testing
ANN1	0.090	0.068
ANN2	0.087	0.063
ANN3	0.085	0.084
ANN4	0.101	0.076
ANN5	0.093	0.096
ANN6	0.098	0.102
ANN7	0.092	0.140
ANN8	0.102	0.080
ANN9	0.100	0.088
ANN10	0.100	0.091
Average	0.095	0.089
Standard deviation	0.006	0.021

#### 5.4.3 Sensitivity analysis

Sensitivity analysis was engaged to evaluate the contribution of each predictor in the development of outcomes, which implies how dependent variables change with independent variables (Sharma et al., 2021). The sensitivity analysis performed in this study aims to measure the predictive power and rank the significance of the antecedents of residents’ acceptance intention toward the reused water. The predicted power of the input neuron is measured by the normalized relative importance of the input constructs on the output constructs. The normalized importance of these input neurons is obtained by dividing the average importance of each input construct by the maximum construct’s average importance and presenting it in the form of a percentage. Table 2 shows the results of the sensitivity analysis which ranks the relative importance of antecedents toward acceptance intention.

**Table 2:** Sensitivity analysis.

Construct	ANN1	ANN2	ANN3	ANN4	ANN5	ANN6	ANN7	ANN8	ANN9	ANN10	Average	Importance	Rank
EE	0.104	0.060	0.046	0.121	0.064	0.050	0.051	0.054	0.203	0.060	8.13%	50.88%	8
FC	0.089	0.059	0.031	0.059	0.014	0.183	0.070	0.182	0.031	0.137	8.55%	53.51%	7
EM	0.210	0.168	0.183	0.137	0.218	0.157	0.188	0.079	0.050	0.207	15.98%	100.00%	1
PC	0.196	0.166	0.164	0.017	0.101	0.097	0.096	0.271	0.223	0.142	14.72%	92.13%	3
SI	0.070	0.076	0.107	0.128	0.088	0.172	0.063	0.022	0.182	0.105	10.14%	63.48%	6
PE	0.142	0.214	0.219	0.176	0.229	0.106	0.212	0.107	0.070	0.083	15.59%	97.61%	2
Cog	0.057	0.051	0.045	0.257	0.215	0.062	0.163	0.204	0.042	0.197	12.94%	81.00%	5
Emo	0.132	0.206	0.205	0.103	0.071	0.172	0.156	0.081	0.199	0.069	13.95%	87.29%	4

PE, Performance Expectancy; EE, Effort Expectancy; FC, Facilitating Conditions; EM, Environmental Motivation; PV, Price Value; SI, Social Influence; CA, Cognitive Attitude; EA, Emotional Attitude.

As presented in Table 2, in terms of the ANN Model, environmental motivation (100.00%) is of great importance to acceptance intention, following by the performance expectancy (97.61%), price value (92.13%), emotional attitude (87.29%), and cognitive attitude (81.00%). The importance rank of the significant factors generated by sensitivity analysis reveals that environmental motivation is the utmost significant predictor of users’ acceptance intention toward the reused water, trailed by the performance expectancy, price value, and attitudes.

## 6 Discussion

Utilizing the UTAUT2 framework, this study is designed to explore the influence of performance expectancy, effort expectancy, social influence, facilitating conditions, environmental motivation, and price value on individuals’ cognitive and emotional attitudes, and the following acceptance intentions. Following the main logic of this study, the discussion section has been structured. The first paragraph of discussion section discusses that how the environmental factors influence cognitive attitudes while the second paragraph discusses the influence of factors on emotional attitudes. At lase, the discussion section explains the influence on final acceptance intention by integrating the SEM and ANN analytical results.

First, this study examined the relationships between the six antecedents and cognitive attitude, while five of these six hypotheses were supported. The results suggest that performance expectancy, effort expectancy, social influence, facilitating conditions, and environmental motivation have statistically significant effects on cognitive attitude. The results are supported by prior literature, such as the work of Smith et al. (2018) and Fielding et al. (2018). Moreover, the analysis results identified effort expectancy as the most significant antecedent. When people can access recycled water easily, conveniently, and effortlessly, they are more likely to develop comprehensive and positive cognitive understandings and evaluations of it (Wester et al., 2016). The finding is in line with the observation of the phenomenon. If recycled water can become an important part of domestic water for residents, the convenient usage of recycled water should be the first consideration (Liu et al., 2018). In contrast, the impact of price value on cognitive attitude is not supported (H6a), though the effect of price value on emotional attitude is significant (H6b). The results unveil that though people may feel delightful and pleasant when they find the price value of recycled water, the price value may not influence their cognitive evaluation of recycled water’s actual value and objective importance. Existing studies mainly explored the relationship between the price value of recycled water and the public acceptance willingness (Chen et al., 2013; Garcia-Cuerva et al., 2016; Iftekhar et al., 2021). This research conducted an exploratory analysis of the influence of price value on individuals’ two-dimensional attitudes and revealed that price value might affect people’s emotional attitudes.

Second, as refers to the emotional attitude, six hypotheses are all supported by empirical evidence. Performance expectancy, effort expectancy, social influence, facilitating conditions, environmental motivation, and price value exert statistically significant effects on emotional attitude. These results are consistent with prior studies (Chen et al., 2013; Fielding et al., 2018; Iftekhar et al., 2021). According to the SEM analysis, we identified the two most significant antecedents are facilitated conditions (*β* = 0.238) and performance expectancy (*β* = 0.211). Previous researchers have highlighted the significance of facilitating conditions offered by governments and water utilities (Hartley et al., 2019; Craddock et al., 2021; Hopson and Fowler, 2022). When the safety and quality of recycled water, information transparency, and responsibility mechanism are ensured, the public is more likely to develop positive emotions toward recycled water, such as trustworthiness and relief (Moya-Fernández et al., 2021; Vila-Tojo et al., 2022). In addition, the importance of performance expectancy is supported by prior literature (Liu et al., 2018). When people perceive the benefits of water reuse, they will be fond of recycled water and develop positive feelings toward (e.g., satisfaction) water reuse (Hurlimann et al., 2008).

Third, as refer to the determinants of acceptance intention, both the SEM and ANN analyses are conducted to offer rich and solid results. The SEM analysis suggests that both cognitive attitude (*β* = 0.339) and emotional attitude (*β* = 0.306) are associated with acceptance intention positively and significantly. The results are in line with the findings in prior studies (Liu et al., 2018; Gao et al., 2019). When residents realize the importance and value of recycled water and develop positive emotions toward reused water, they are more likely to make the acceptance decision (Ding et al., 2022).

In addition, the ANN analysis is performed to unveil the importance rank of all the antecedents of acceptance intention. Due to the complexity of individuals’ decision-making processes regarding recycled water, the relationships between the factors and behavior might not necessarily be linear (Chong, 2013). The SEM analysis, as a singular linear analysis, could oversimplify the complexity of such decisions. The ANN analysis endeavors to compensate for the SEM analysis and offer a comprehensive understanding of the phenomenon of interest (Wang et al., 2022). The results unveil two interesting findings. First, the ANN results verified the analytical results of SEM and indicated that cognitive attitude (81.00%) and emotional attitude (87.29%) are important predictors in facilitating residents’ acceptance intention of recycled water. Second, the ANN analysis results identify the top three critical determinants of individuals’ acceptance intention, including environmental motivation, performance expectancy, and price value. The SEM results discovered the linear associations between these factors and two dimensions of attitude, while ANN analysis revealed the significance of these factors in determining acceptance intention. The ANN results suggest that individuals’ acceptance of recycled water is mainly motivated by people’s motivation for environmental protection, the benefits of using recycled water, and price value. In other words, compared to others’ opinions (social influence), the policy, information, and responsibility mechanism (facilitating condition), and convenience of the water facility (effort expectancy), residents’ decisions are determined by the environmental, utilitarian, and monetary benefits at a greater extent.

## 7 Conclusion

Based on the findings of residents’ recycled water acceptance intention, the following subsections outline the contributions to theory development, summarize suggestions for practical implications, and describe limitations and future orientations regarding the research.

### 7.1 Theoretical implication

This study endeavors to contribute multiple theoretical implications to the existing body of literature. To our knowledge, it stands as one of the pioneering efforts in employing the technology adoption perspective to interpret public acceptance of recycled water. Most extant studies identified psychological, social, and sociodemographic factors and investigated the phenomenon from a social and psychological perspective (Chen et al., 2013; Hartley et al., 2019; Hou et al., 2020; Leong and Lebel, 2020; Ding et al., 2022). Our research unveils that the technology adoption perspective offers a solid theoretical framework to not only comprehensively investigate the significant diversified factors influencing people’s perceptions of recycled water, but also contribute to the understanding of the features of recycled water as a technological product.

Second, this study applies the UTAUT2 framework in the context of recycled water acceptance to illustrate the complexity of people’s decision-making process toward recycled water. We extend the application of UTAUT2 by integrating the individual’s cognitive and emotional reactions into the research model and verify the proposed model with empirical evidence in the field of recycled water. Hence, this research not only contributes to the recycled water literature by investigating the complexity of individuals’ acceptance processes based on the technological theoretical perspective but also provides useful knowledge to UTAUT2 literature in an emerging area.

Last but not least, we employ a hybrid SEM-ANN approach to address the complexity of individuals’ decision-making processes. This study is one of the first to apply the analytical design of SEM-ANN to explore the acceptance intention in the field of recycled water. The SEM and ANN analytical techniques validate and complement each other by confirming the hypotheses proposed in the research model and unveiling the importance rank of the critical determinants of acceptance of recycled water with a higher predictive power. Thus, this research contributes to an emerging and complex field of recycled water by offering rich and comprehensive interpretations of this phenomenon with a mixed analytic design.

### 7.2 Practical implication

The research results provide several implications for recycled water policymakers and water utilities. First, the SEM analysis results identified three factors that are important to either cognitive attitude or emotional attitude, which are strong determinants of recycled water acceptance. Recycled water policymakers and water utilities should especially allocate resources to improve these factors, including effort expectancy, facilitating conditions, and performance expectancy. As refer to effort expectancy, the water supply system should be distributed fairly to guarantee that people can access recycled water easily, conveniently, and effortlessly. In addition, facilitating measures should be adopted to encourage residents to accept recycled water, such as ensuring information transparency and designing the responsibility mechanism. At last, more tremendous efforts should also be made to publicize and popularize the usefulness of recycled water.

Second, ANN analysis has revealed two other factors with the predictive power of the acceptance intention of recycled water, namely environmental motivation and price value. The recycled water policymakers should enhance people’s environmental motivation in multiple ways, such as broadcasting the news about the severity of water shortages and organizing community activities to propagate the environmental hazards of water scarcity. In addition, water utilities should extensively investigate the price range of recycled water preferred by the masses and set an affordable price. At the same time, the reward mechanisms should be designed carefully to stimulate residents’ acceptance of recycled water.

### 7.3 Limitations and future study

This research aims to provide valuable insights to both academic literature and practitioners in the field of recycled water acceptance, while there still exists limitations for future study to address. First, from the methodological and sampling perspective, this study may have some limitations. We recruited the survey sample from China due to the severity of water shortages in China. The geographical and cultural representativeness of the study maybe limited, which potentially constrains the applicability of the research findings across different regions or cultural contexts. Future studies should explore this focal phenomenon in other countries or regions with different levels of regional development, different cultures, economic situations, and social environments. In addition, our research employed the cross-sectional survey to collect the empirical data at a point in time and applied a mixed analytical approach to analyze the empirical data. Hence, future studies may adopt other research designs to collect and analyze empirical data and examine different research objectives (e.g., actual behavior), such as the qualitative interview, empirical experiment, and comparative analysis, to offer a robust research design and comprehensive methodological approaches to the phenomenon. Moreover, most of the respondents in our study are younger users, future studies can further conduct the comparative analysis of the acceptance intention between younger and elderly people.

Secondly, though this study attempted to capture comprehensive factors to represent the predicting factors in this context, this study did not incorporate all the possible antecedents in the research model. Hence, future studies can investigate more critical influential determinants from diverse perspectives. In addition, UTAUT2 researchers have called the examinations on the moderating role of sociodemographic and cultural characteristics in determining individuals’ behaviors (Baptista and Oliveira, 2015; Hew et al., 2015).

## Data availability statement

The original contributions presented in the study are included in the article/Supplementary material, further inquiries can be directed to the corresponding authors.

## Ethics statement

The studies involving humans were approved by School of Finance and economics, Xi’an Jiaotong University. The studies were conducted in accordance with the local legislation and institutional requirements. The participants provided their written informed consent to participate in this study.

## Author contributions

X-YX: Writing – review & editing, Writing – original draft, Supervision, Investigation, Conceptualization. Y-BH: Writing – original draft, Resources, Project administration, Investigation, Funding acquisition. Y-XG: Writing – original draft, Validation, Investigation. Q-DJ: Writing – review & editing, Writing – original draft, Software, Methodology, Conceptualization.

## Footnotes

^1^https://www.sojump.com/
