# Supplementary material for: Exploring the recycled water acceptance based on the technological perspective of UTAUT2: a hybrid analytical approach
Source: Front Psychol. 2024 Jun 18;15:1384635. doi: 10.3389/fpsyg.2024.1384635 (PMC11217519; doi:10.3389/fpsyg.2024.1384635)
Supplement: Supplementary file 1 [file Table_1.PDF]

Appendix

Appendix A. Measurements.

Constructs		Measurement items	References
Performance Expectancy (PE)		I think it's important for people to use recycled water (RW) because	Venkatesh et al. (2012); Khan et al. (2017); Liu et al. (2018)
	PE1	it is useful in their daily life taking to light the environmental concerns of water shortage.	
	PE2	it can increase people's chances of achieving things that are important to them such as conserving resources.	
	PE3	it can reduce the consumption of water resources as it serves as an alternative water source.	
	PE4	it can protect our environment by reducing wastewater discharged to the environment.	
	PE5	it is useful to create a better environment for our future generations as it preserves water.	
Effort Expectancy (EE)		I think it is important for people to	Venkatesh et al. (2012); Khan et al. (2017); Liu et al. (2018)
	EE1	conveniently use RW if they want to.	
	EE2	be well informed about RW to the extent of being able to distinguish facilities using RW.	
	EE3	have the option to use RW.	
	EE4	use recycled water as long as they want to use it.	
Social Influence (SI)		I think	Venkatesh et al. (2012)
	SI1	role models can influence people to use RW.	
	SI2	family and friends can motivate people to use RW.	
	SI3	people use of RW because it has a status symbol in our modern-day society, it conveys water literacy.	

**Constructs**		**Measurement items**	**References**
	SI4	people use RW because it has a positive image in the society as it relays environmental awareness.	
	SI5	people use RW because it reflects their preference of being proactive with water conservation.	
Facilitating Conditions (FC)		I think it is important for people to	Brown and Venkatesh (2005); Venkatesh et al. (2012)
	FC1	have the knowledge about water literacy, and motivation to take action to conserve and protect fresh water resource.	
	FC2	have easy access to information about RW from others if they have any doubts.	
	FC3	have the government supports the use of RW.	
	FC4	trust the authorities to ensure the safety of the RW.	
Price Value (PV)		I think it is important for RW to	Venkatesh et al. (2012)
	PV1	be reasonably priced.	
	PV2	be a good value for the money.	
	PV3	to provide a good value.	
	PV4	have an affordable price.	
Environmental Motivation (EM)		I think it is important for people to consider using RW because	Venkatesh et al. (2012); Razak et al. (2014)
	EM1	it does not harm the environment but rather preserves it.	
	EM2	it helps save the environment for future generations.	
	EM3	it causes less pollution thus preserving the environment.	
	EM4	people have a moral obligation to protect the environment.	
Emotional Attitude (EA)		I feel	Chui et al. (2019);
	EA1	good when I use RW.	

Constructs		Measurement items	References
	EA2	enthusiastic to use RW.	Zhang et al. (2021)
	EA3	honored if I could use RW.	
	EA4	interested in using RW.	
Cognitive Attitude (CA)	CA1	It means a lot to me to use RW.	Chui et al. (2019); Zhang et al. (2021)
	CA2	It is valuable for me to use RW.	
	CA3	It is relevant to me to use RW.	
	CA4	I am knowledgeable about RW.	
Acceptance Intention (AI)		I think	Venkatesh et al. (2012); Khan et al. (2017)
	AI1	many people would consider it important to continue using RW in the future.	
	AI2	many people would consider it important to always try to use RW in their daily life.	
	AI3	many people would consider planning to continue using RW frequently.	
	AI4	many people would consider it important to recommend others people to save water and use RW.	

Appendix B. Demographics (N=308).

Variable	Category	Count	Percentage
Gender	Male	147	47.7%
	Female	161	52.3%
Age	20-25	65	21.1%
	26-30	93	30.2%
	31-35	71	23.1%

	36-40	44	14.3%
	41-50	26	8.4%
	Above 50	7	2.9%
Education	Below primary level	15	4.9%
	Junior middle school	28	9.1%
	High School	72	23.4%
	College	162	52.6%
	University	31	10.1%
Income per month (¥)	≤2000	72	13.2%
	2001~5000	180	39.0%
	5001~8000	213	33.0%
	Above 80000	81	13.8%

Appendix C. Reliability and Convergent Validity Analysis.

Constructs	Items	Factor Loading	Composite reliability	Cronbach's Alpha	Convergence (AVE)
Performance Expectancy (PE)	PE1	0.805***	0.905	0.868	0.655
	PE2	0.816***			
	PE3	0.781***			
	PE4	0.833***			
	PE5	0.811***			
Effort Expectancy (EE)	EE1	0.831***	0.900	0.852	0.663
	EE2	0.866***			
	EE3	0.815***			
	EE4	0.818***			
Social Influence (SI)	SI1	0.743***	0.866	0.807	0.613
	SI2	0.736***			
	SI3	0.720***			
	SI4	0.798***			
	SI5	0.759***			

Constructs	Items	Factor Loading	Composite reliability	Cronbach's Alpha	Convergence (AVE)
Facilitating Conditions (FC)	FC1	0.772***	0.874	0.808	0.618
	FC2	0.777***			
	FC3	0.824***			
	FC4	0.811***			
Price Value (PV)	PV1	0.789***	0.905	0.869	0.657
	PV2	0.843***			
	PV3	0.809***			
	PV4	0.789***			
Environmental Motivation (EM)	EM1	0.814***	0.897	0.846	0.757
	EM2	0.809***			
	EM3	0.782***			
	EM4	0.818***			
Emotional Attitude (EA)	EA1	0.863***	0.905	0.860	0.693
	EA2	0.836***			
	EA3	0.843***			
	EA4	0.863***			
Cognitive Attitude (CA)	CA1	0.837***	0.913	0.873	0.600
	CA2	0.861***			
	CA3	0.856***			
	CA4	0.801***			
Acceptance Intention (AI)	AI1	0.740***	0.858	0.780	0.602
	AI2	0.809***			
	AI3	0.792***			
	AI4	0.761***			

Appendix D. Fornell-Larcker Criterion**.**

Construct	PE	EE	SI	FC	EM	PV	CA	EA	AI
PE	0.809								
EE	0.266	0.832							
SI	0.215	0.399	0.752						
FC	0.264	0.259	0.311	0.796					
EM	0.227	0.364	0.179	0.150	0.811				
PV	0.247	0.186	0.237	0.266	0.151	0.807			
CA	0.337	0.427	0.334	0.345	0.312	0.265	0.839		
EA	0.413	0.398	0.310	0.411	0.314	0.365	0.485	0.851	

AI	0.264	0.291	0.334	0.229	0.268	0.189	0.320	0.312	0.776

Note: Squared correlations; the square root of AVE in the diagonal.

Appendix E. Inter-Construct Correlations**.**

Construct	PE	EE	SI	FC	EM	PV	CA	EA	AI
PE	1.000								
EE	0.516	1.000							
SI	0.463	0.632	1.000						
FC	0.514	0.509	0.558	1.000					
EM	0.477	0.603	0.423	0.387	1.000				
PV	0.497	0.432	0.487	0.515	0.388	1.000			
CA	0.581	0.653	0.578	0.587	0.559	0.515	1.000		
EA	0.642	0.630	0.557	0.640	0.560	0.604	0.696	1.000	
AI	0.514	0.540	0.578	0.478	0.517	0.435	0.565	0.559	1.000

Appendix F. Abbreviations

**Abbreviations**	**Full Names**
PE	Performance Expectancy
EE	Effort Expectancy
SI	Social Influence
FC	Facilitating Conditions
EM	Environmental Motivation
PV	Price Value

CA	Cognitive Attitude
EA	Emotional Attitude
AI	Acceptance Intention
